# Oropouche infection a neglected arbovirus in patients with acute febrile illness from the Peruvian coast

**DOI:** 10.1186/s13104-020-4937-1

**Published:** 2020-02-10

**Authors:** Johanna Martins-Luna, Juana del Valle-Mendoza, Wilmer Silva-Caso, Isabel Sandoval, Luis J. del Valle, Carlos Palomares-Reyes, Hugo Carrillo-Ng, Isaac Peña-Tuesta, Miguel Angel Aguilar-Luis

**Affiliations:** 1 Research and Innovation Centre of the Faculty of Health Sciences, Universidad Peruana de Ciencias Aplicadas, Av. San Marcos Cuadra 2, Chorrillos, Lima, Peru; 2 School of Medicine, Faculty of Health Sciences, Universidad Peruana de Ciencias Aplicadas, Lima, Peru; 3 Laboratorio de Biología Molecular, Instituto de Investigación Nutricional, Lima, Peru; 4 Comité del Médico Joven-Consejo Nacional, Colegio Médico del Perú, Lima, Peru; 5 Red de Salud de Morropón Chulucanas, Dirección Regional de Salud de Piura (DIRESA-Piura), Piura, Peru; 6 Barcelona Research Center for Multiscale Science and Engineering, Departament d’Enginyeria Química, EEBE, Barcelona Universitat Politècnica de Catalunya (UPC), Barcelona, Spain

**Keywords:** Peru, Arbovirus, Oropouche, Dengue, Chikungunya, Zika, PCR

## Abstract

**Objective:**

To evaluate the frequency of infection caused by the Oropouche virus (OROV) in 496 patients with acute febrile disease (AFI), whose samples were obtained for the analysis of endemic arboviruses in a previous investigation carried out in 2016.

**Results:**

OROV was detected in 26.4% (131/496) of serum samples from patients with AFI. Co-infections with Dengue virus (7.3%), Zika virus (1.8%) and Chikungunya (0.2%) were observed. The most common clinical symptoms reported among the patients with OROV infections were headache 85.5% (112/131), myalgia 80.9% (106/131), arthralgia 72.5% (95/131) and loss of appetite 67.9% (89/131). Headache and myalgia were predominant in all age groups. Both OROV infections and co-infections were more frequent in May, June and July corresponding to the dry season of the region.

## Introduction

The epidemiological characteristics of arthropod-borne viral infections (arbovirosis) are changing worldwide. This represents a serious threat to public health due to the presence of emerging and/or reemerging viruses with high epidemic potential [1]. Arboviruses such as the Dengue virus (DENV), the Yellow fever virus (YFV), West Nile virus (WNV) among other viruses are endemic in the region and can, unpredictably, cause new epidemics [2, 3].

Currently, the appearance and dispersion of arboviruses are faster and encompass larger areas [3]. This due to factors that interact with each other, related to the virus, humans, and the environment (temperature, humidity, precipitation, etc.). These factors affect the spatial and temporal distribution, as well as the abundance of the arthropods, the characteristics of the life cycles and the transmission efficiency [4]. Additionally, the implementation of new molecular techniques has also contributed to a more accurate etiological diagnosis in undifferentiated acute febrile illnesses (AFI) [5, 6].

In Peru situation is similar, studies have reported the presence of the four serotypes of DENV in the Peruvian coast, being the serotype DENV 2 the most frequent during the period from May to August (i.e., in 2016), as well as the Zika virus (ZIKV) and the Chikungunya virus (CHIKV) [7]. In the last 3 years, the presence of the Oropouche virus (OROV) has been reported, reemerging in some areas such as Madre de Dios or recently appearing in other areas such as Huánuco [8, 9]. Our study describes the presence of OROV, an emerging pathogenic arbovirus in the north coast of Peru and its main clinical characteristics.

## Main text

### Methods

#### Patients and sampling

This study was performed using samples stored in our biobank during the months of February to September 2016. The primary study was conducted in Piura, coastal region in northwestern Peru, that has been recognized as an endemic area for Dengue and other arboviral etiologies with low laboratory confirmation rates [10–12]. In the first study, 496 samples from patients with Acute Febrile DiseaseIllness (AFI) were analyzed and DENV, CHIKV, or ZIKV were identified in 46.8% (232/496) of the samples. The current study was performed including the total number (496 samples) of patients who met the criteria of AFI (axillary temperature ≥ 38 °C for less than 7 days together with one or more the signs and/or symptoms associated with arbovirus infections described in previous works and developed later in the text [7, 13]. We excluded samples in inadequate state of conservation, samples without codification, improperly filled data sheets and those with incomplete demographic data such as age, gender, place of origin, etc.

#### Ethics statement

Approved by the Research Ethics Board of the Hospital Regional de Cajamarca, Peru. The samples were obtained in the context of the epidemiological/syndromic surveillance program according to the health directives of the National Center for Epidemiology, Disease Control Prevention of the Ministry of Health of Peru. In this way, the collection of samples was exempt of informed consent.

#### Samples

One serum sample per patient was collected by using Vacuette^®^ TUBE Serum Separator Clot Activator (Vacuette, Kremsmünster, Austria); all the samples were stored at − 80 °C.

#### Molecular detection of OROV, DENV, CHIKV, and ZIKV

RNA extraction was performed from 200 μL of serum samples, RNA was extracted with the High Pure RNA Isolation Kit (Roche Applied Science, Mannheim, Germany). Then, cDNA was synthethized from 2.5 μL of RNA, using the Transcriptor First Strand cDNA Synthesis kit (Roche Life Science, Mannheim, Germany), according to the manufacturer’s instructions.

Amplification by PCR assay for the detection of OROV was carried out using the primers described by Moreli et al. [14], and PCR conditions described by Silva-Caso et al. [9].

Amplification by Real-time RT-PCR assay for DENV, CHIKV, and ZIKV was performed with the primers and the probe used for DENV, CHIKV, and ZIKV described by Leparc-Goffart et al. [15], Panning M et al. [16] and Faye et al. [17], respectively. The PCR conditions were described by Sánchez-Carbonel et al. [7].

The control RNA was also provided by Centers for Disease Control and Prevention (CDC, Fort Collins, Colorado, USA). An internal control reaction was run for each of the samples as mentioned by the CDC instructions to confirm the integrity of the extraction reagents and the successful recovery of RNA. PCR products were purified using SpinPrepTM Gel DNA Kit, San Diego, USA and sequenced by Sanger method (Macrogen, Seoul, Korea).

#### Data analysis

A database according to the study was managed in the excel software (Microsoft). Qualitative variables were reported as frequencies in percentages. χ^2^-test was used to determine the distribution differences between groups. Fisher’s exact test (F-test) was used to compare one or two proportions. All analyses were processed with the Minitab Inc. software v18.1 (USA). The graphic representation of the data was made with the OriginPro v10 software (OriginLab Corp., USA).

### Results

**Fig. 1:**
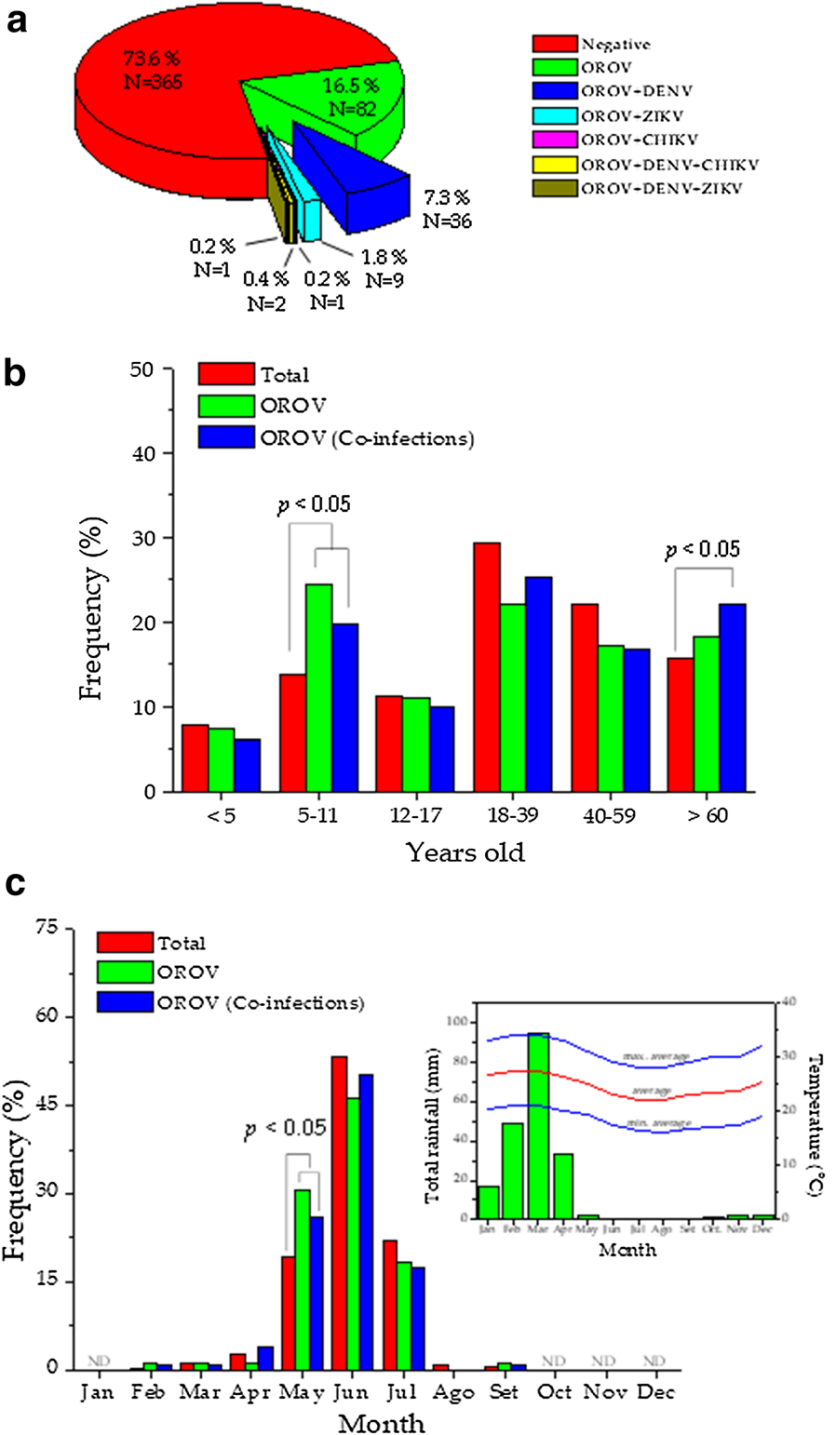
Frequency of infection with OROV and OROV + other arboviruses (co-infections) (**a**). Age distribution of infected patients (**b**). Seasonality of infection with OROV and its co-infections (**c**); the inset shows annual rainfall and average temperature data with its maximum and minimum [31]

OROV was detected in 26.4% (131/496) of AFI patients. We have that 16.5% (82/496) (95% CI 13.4–20.1%) of AFI patients were diagnosed with OROV as a single infectious agent and co-infection of OROV with other arboviruses was identified in 9.9% (49/496) (95% CI 7.4–12.8%). Co-infections of two viruses were the most frequent, such as OROV + DENV (36 cases), OROV + ZIKV (9 cases) and OROV + CHIKV (1 case). In addition, 3 cases of co-infection with three viruses simultaneous arboviruses were also identified (2 cases of OROV + DENV + CHIKV, and 1 case of OROV + DENV + ZIKV) (see Fig. 1a, Table 1). Both cases (infections by OROV and OROV + other arboviruses) were significant (p < 0.05) in the AFI patient population. This highlights the importance of co-infections as an epidemiological condition in the population.

**Table 1:** Demographic characteristics of patients with OROV and co-infections

Characteristic	Total, n = 496 (%)	Negatives, n = 365 (%)	OROV, n = 131 (%)	Co-infections				
				OROV, n = 82 (%)	OROV/DENV, n = 36 (%)	OROV/ZIKV, n = 9 (%)	OROV/CHIKV, n = 1 (%)	Others**, n = 3 (%)
Age (years)								
< 5	39 (7.9)	31 (8.5)	8 (6.1)	6 (7.3)	2 (5.6)	0 (0.0)	0 (0.0)	0 (0.0)
5–11	68 (13.7)	42 (11.5)	26 (19.8)*	20 (24.4)*	6 (16.7)	0 (0.0)	0 (0.0)	0 (0.0)
12–17	56 (11.3)	43 (11.8)	13 (9.9)	9 (11.0)	1 (2.8)	3 (33.3)	0 (0.0)	0 (0.0)
18–39	146 (29.4)	113 (31.0)	33 (25.2)	18 (22.0)	9 (25.0)	3 (33.3)	1 (100.0)	2 (66.7)
40–59	109 (22.0)	87 (23.8)	22 (16.8)	14 (17.1)	5 (13.9)	2 (22.2)	0 (0.0)	1 (33.3)
≥ 60	78 (15.7)	49 (13.4)	29 (22.1)*	15 (18.3)	13 (36.1)*	1 (11.1)	0 (0.0)	0 (0.0)
*χ*^*2*^-test, *p* value	0.823		0.017	0.029	0.006	ND	ND	ND
F-test, *p*-value			0.025	0.004	0.001			
Gender								
Male	226 (45.6)	154 (42.2)	72 (55.0)	43 (52.4)	24 (66.7)	4 (44.4)	0 (0.0)	1 (33.3)
Female	270 (54.4)	211 (57.8)	59 (45.0)	39 (47.6)	12 (33.3)	5 (55.6)	1 (100.0)	2 (66.7)
F-test, *p*-value	0.006	< 0.001	0.138	0.640	0.009	ND	ND	ND

χ^2^-test, negative vs total and infected cases. *ND* not determined because there are many cells with counts less than 5

*F-test, negative vs infected cases. **Others correspond to infection cases by OROV + DENV + ZIKV and OROV + DENV + CHIKV

An analysis of the infection was performed by age groups (Fig. 1b, Table 1). The population of AFI patients without infection caused by OROV were considered as negative cases. The distribution was significantly different in those infected only by OROV (χ^2^, p = 0.029) and those with OROV + DENV co-infection (χ^2^, p = 0.006). Consequently, the total population of patients infected with OROV (OROV and OROV + other arboviruses) was significant (χ^2^, p = 0.017) compared to negative cases. This difference is based on the significant increase (p < 0.025) of the OROV positive cases in the groups of children aged 5–11 years and adults older than 60 years.

Regarding the analysis of sex and the distribution of infection (Table 1). Patients infected only with OROV showed a higher non-significant frequency of males (52.4%), but in the case of OROV + DENV co-infection the frequency of males (66.7) was highly significant (p < 0.009).

The frequency of OROV infection was also analyzed for its possible seasonality (Fig. 1c, Additional file 1: Table S1). The annual distribution of the frequencies of OROV infection and co-infection of OROV + other arboviruses were significantly different (χ^2^ with p = 0.045 and p = 0.016, respectively) to the distribution of AFI and negative cases. The distribution is unimodal between the months of May and July and the greatest number of cases of infection with ORV and OROV with co-infections (46.3% and 50.4%, respectively) is observed in the month of June. We found that OROV infection mainly occurs during late autumn and mid-winter. However, if we observe the annual distribution of rainfall and the little variation in temperature in the geographical area of study (inset Fig. 1c), the rainy season corresponds to summer-autumn. In this sense, it is more accurate to indicate that OROV infection occurs mostly in the dry season.

In reference to the clinical presentation. The analysis of the distribution of clinical symptoms does not show significant differences (χ^2^, p > 0.05) to differentiate AFI conditions from OROV infections and OROV + other arbovirus co-infections (Table 2, Additional file 1: Table S2). This means that it is very difficult to diagnose OROV infection clinically, and confirmatory molecular diagnosis is required. In all cases of co-infections, the frequency in the clinical presentation was similar to that presented in the cases only positive OROV (Table 2). When describing the clinical picture of OROV + patients, it was found that headache and myalgia are the most frequent in all age groups (Additional file 1: Table S3).

**Table 2:** Clinical presentation of patients with OROV

	Total, n = 496 (%)	OROV, n = 131 (%)	Co-infections				
			OROV, n = 82 (%)	OROV/DENV, n = 36 (%)	OROV/ZIKV, n = 9 (%)	OROV/CHIKV, n = 1 (%)	Others*, N = 3 (%)
Clinical symptoms							
Headache	444 (89.5)	112 (85.5)	71 (86.6)	29 (80.6)	8 (88.9)	1 (100.0)	3 (100.0)
Myalgia	419 (84.5)	106 (80.9)	66 (80.5)	27 (75.0)	9 (100.0)	1 (100.0)	3 (100.0)
Arthralgia	396 (79.8)	95 (72.5)	57 (69.5)	28 (77.8)	7 (77.8)	1 (100.0)	2 (66.6)
Retroocular pain	337 (67.9)	70 (53.4)	44 (53.7)	17 (47.2)	6 (66.7)	1 (100.0)	2 (66.6)
Hyporexia	312 (62.9)	89 (67.9)	57 (69.5)	21 (58.3)	7 (77.8)	1 (100.0)	3 (100.0)
Low back pain	270 (54.4)	66 (50.4)	37 (45.1)	19 (52.8)	7 (77.8)	1 (100.0)	2 (66.6)
Nausea/Vomiting	251 (50.6)	62 (47.3)	40 (48.8)	15 (41.7)	5 (55.6)	0 (0.0)	2 (66.6)
Odynophagia	184 (37.1)	48 (36.6)	33 (40.2)	11 (30.6)	3 (33.3)	0 (0.0)	1 (33.3)
Acne	89 (17.9)	25 (19.1)	15 (18.3)	7 (19.4)	3 (33.3)	0 (0.0)	0 (0.0)
Shaking chills	3 (0.6)	0 (0.0)	0 (0.0)	0 (0.0)	0 (0.0)	0 (0.0)	0 (0.0)
Conjuntival injection	3 (0.6)	1 (0.8)	0 (0.0)	1 (2.8)	0 (0.0)	0 (0.0)	0 (0.0)
Dizziness	1 (0.2)	0 (0.0)	0 (0.0)	0 (0.0)	0 (0.0)	0 (0.0)	0 (0.0)
Cough	1 (0.2)	0 (0.0)	0 (0.0)	0 (0.0)	0 (0.0)	0 (0.0)	0 (0.0)
Bleeding manifestations							
Petechiae	11 (2.2)	1 (0.8)	1 (1.2)	0 (0.0)	0 (0.0)	0 (0.0)	0 (0.0)
Epistaxis	9 (1.8)	0 (0.0)	0 (0.0)	0 (0.0)	0 (0.0)	0 (0.0)	0 (0.0)
Gingivorrhagia	3 (0.6)	0 (0.0)	0 (0.0)	0 (0.0)	0 (0.0)	0 (0.0)	0 (0.0)
Ecchymosis	2 (0.4)	0 (0.0)	0 (0.0)	0 (0.0)	0 (0.0)	0 (0.0)	0 (0.0)
Mane	2 (0.4)	2 (1.5)	2 (2.4)	0 (0.0)	0 (0.0)	0 (0.0)	0 (0.0)
Gynecrosis	1 (0.2)	1 (0.8)	1 (1.2)	0 (0.0)	0 (0.0)	0 (0.0)	0 (0.0)
Hemoptoic sputum	1 (0.2)	1 (0.8)	1 (1.2)	0 (0.0)	0 (0.0)	0 (0.0)	0 (0.0)
Alarm signals							
Intense and continuous abdominal pain	22 (4.4)	4 (3.1)	4 (4.9)	0 (0.0)	0 (0.0)	0 (0.0)	0 (0.0)
Platelet Decrease	7 (1.4)	1 (0.8)	1 (1.2)	0 (0.0)	0 (0.0)	0 (0.0)	0 (0.0)
Persistent vomiting	6 (1.2)	3 (2.3)	2 (2.4)	1 (2.6)	0 (0.0)	0 (0.0)	0 (0.0)
Hematocrit Increase	6 (1.2)	1 (0.8)	1 (1.2)	0 (0.0)	0 (0.0)	0 (0.0)	0 (0.0)
Chest pain or dyspnea	5 (1.0)	1 (0.8)	1 (1.2)	0 (0.0)	0 (0.0)	0 (0.0)	0 (0.0)
Sudden decrease in T° or hypothermia	3 (0.6)	1 (0.8)	1 (1.2)	0 (0.0)	0 (0.0)	0 (0.0)	0 (0.0)
Excessive decay or lipotimia	3 (0.6)	1 (0.8)	1 (1.2)	0 (0.0)	0 (0.0)	0 (0.0)	0 (0.0)
Altered mental state (drowsiness)	1 (0.2)	0 (0.0)	0 (0.0)	0 (0.0)	0 (0.0)	0 (0.0)	0 (0.0)
Hepatomegaly or jaundice	1 (0.2)	0 (0.0)	0 (0.0)	0 (0.0)	0 (0.0)	0 (0.0)	0 (0.0)
Crash signs							
Cold or cyanotic limbs	2 (0.4)	1 (0.8)	1 (1.2)	0 (0.0)	0 (0.0)	0 (0.0)	0 (0.0)
Arterial hypotension	1 (0.2)	1 (0.8)	1 (1.2)	0 (0.0)	0 (0.0)	0 (0.0)	0 (0.0)
Fast and weak pulse	1 (0.2)	1 (0.8)	1 (1.2)	0 (0.0)	0 (0.0)	0 (0.0)	0 (0.0)
BP differential < 20 MMHg	1 (0.2)	0 (0.0)	0 (0.0)	0 (0.0)	0 (0.0)	0 (0.0)	0 (0.0)

### Discussion

OROV is not traditionally known as one of the five most common arboviruses emerged in the last century leading to potential neglect of its burden [3, 4, 18, 19]. In the last 4 years, several outbreaks of Oropouche fever have been reported in Peruvian rural and urban communities in the Amazon [8, 20]. However, no official reports of OROV have been published yet about other areas affected apart from the Peruvian amazon, except from a 2011 report in Cajamarca that is located northern highlands, in the Andes Mountains [21].

Due to the high number of negative samples, in our previous study, we pursued OROV detection given that the genus Culicoides and Culex mosquitoes, which are the main vector for Oropouche fever, have been previously described in the region [22–24]. Surprisingly, OROV was detected in 26.4% (131/496) of cases and co-infections with DENV, ZIKV virus and CHIKV virus were also reported. This finding suggests that OROV was the second most prevalent arbovirus diagnosed in the study population [7].

In Peru, it has been suggested that OROV spreading across the riverbanks of the Amazon River is facilitated by human mobilization [25]. Moreover, in 2016 an eco-epidemiological assessment of the Oropouche fever outbreak in Cusco, Peru suggested patterns of vegetation loss in the study area which could help explain outbreak occurrence [26].

The present study is the first report of OROV detection in the Peruvian coastal region demonstrating a high incidence of this arbovirus in samples negatives for DENV, CHIKV, and ZIKV in a first analysis. We suspect that landscape perturbation in addition to human migration may have played a key role in the virus spread probably from Cajamarca which part of its eastern territory includes the Amazon Rainforest and limits on the west with Piura.

Infections in humans caused by OROV are characterized as an acute febrile illness, usually accompanied by headache, myalgia, arthralgia, anorexia, dizziness, chills, and photophobia [18]. These clinical symptoms are observed in around 60% of patients resembling those of classical arboviral infection and highlighting the importance of laboratory diagnostic test [26]. This non-specific presentation has been also observed in our population where headache, myalgias and arthralgias were among the most common symptoms across DENV, CHIKV, ZIKV and OROV samples, with no differences even in patients with co-infections between these arboviruses [7].

The epidemiological surveillance of OROV is based on acute phase serology (IgM) [19, 27]. Serological tests depend on several factors, being the timing of sample collection stands out. Sampling is recommended 5 days after the onset of symptoms in order to detect immunoglobulins in viremia peaks [19]. In Peru surveillance is done only with serological testing and limited only to regions in which the virus is endemic [28].

Our work shows an important frequency of co-infections among the arboviruses studied, this idea is reinforced with studies that have shown that the vector *Aedes aegypti* can transmit both ZIKV and CHIKV through a single bite and that the co-infection of ZIKV and CHIKV does not influence its vectorial competence [29], which could also be applied for DENV. It has also been described that *Aedes aegypti* and *Aedes albopictus* can transmit all combinations of ZIKV, DENV, and CHIKV to humans, our report suggests that there may be co-infection with OROV although transmitted by different vector. In this context, it has been studied that simultaneous co-infection between 2 or 3 different viruses alters the immune response but that this does not imply a different or more serious clinical symptomatology if it is contrasted with single viral infections [29, 30].

In conclusion, we believe that Oropouche fever should be included in the febrile syndromes surveillance system in Peru. This is based on the cocirculation of multiple arboviruses and the similarity of clinical symptoms.

## Limitations

The study design does not allow establishing the causality between the positive samples for OROV and the clinical presentation of the disease. The patient was not followed up due to recruitment in outpatient health facilities. The reorganization of the virus was not evaluated due to our limited resources, however, we propose its evaluation in future work on this virus.

## Supplementary information

**Additional file 1: Table S1.** Positive cases of arbovirus and seasonality. χ^2^-test, negative cases vs total cases and infected. χ^2^-test was performed for the May–July period to avoid counts less than 5. ND, not determined because there are many cells with counts less than 5. *F-test, negative cases vs infected. **Others correspond to infection cases by OROV/DENV/ZIKV and OROV/DENV/CHIKV. **Table S2.** Analysis of the distribution of clinical symptoms. The table is symmetric on the diagonal line. The numbers correspond to the *p*-values associated with χ^2^-Test, no significant differences (*p* > 0.05). ND, not determined because there are many cells with counts less than 5. *Others correspond to infection cases by OROV + DENV + ZIKV and OROV + DENV + CHIKV. **Table S3.** Positive cases of OROV by age and symptomatology. χ^2^-test, total positive cases vs positive cases by age.

## Data Availability

Abstraction format used in the study and dataset are available and accessible from the corresponding author upon request in the link: https://figshare.com/articles/Dataset_Piura/5766000.

## Abbreviations

OROV: Oropouche virus
PCR: Polymerase chain reaction
DNA: Deoxyribonucleic acid
bp: Base pairs
