# Supplementary material for: Oropouche infection a neglected arbovirus in patients with acute febrile illness from the Peruvian coast
Source: BMC Res Notes. 2020 Feb 10;13:67. doi: 10.1186/s13104-020-4937-1 (PMC7011230; doi:10.1186/s13104-020-4937-1)
Supplement: Supplementary file 1 — Additional file 1: Table S1. Positive cases of arbovirus and seasonality. χ2-test, negative cases vs total cases and infected. χ2-test was performed for the May–July period to avoid counts less than 5. ND, not determined because there are many cells with counts less than 5. *F-test, negative cases vs infected. **Others correspond to infection cases by OROV/DENV/ZIKV and OROV/DENV/CHIKV. Table S2. Analysis of the distribution of clinical symptoms. The table is symmetric on the diagonal line. The numbers correspond to the p-values associated with χ2-Test, no significant differences (p > 0.05). ND, not determined because there are many cells with counts less than 5. *Others correspond to infection cases by OROV + DENV + ZIKV and OROV + DENV + CHIKV. Table S3. Positive cases of OROV by age and symptomatology. χ2-test, total positive cases vs positive cases by age. [file 13104_2020_4937_MOESM1_ESM.docx]

**Table S1:** Positive cases of arbovirus and seasonality. χ^2^-test, negative cases vs total cases and infected. χ^2^-test was performed for the May-July period to avoid counts less than 5. ND, not determined because there are many cells with counts less than 5. *F-test, negative cases vs infected. **Others correspond to infection cases by OROV+DENV+ZIKV and OROV+DENV+CHIKV.

| **MONTH** | **Total**  **n=496 (%)** | **Negatives**  **n= 365 (%)** | **OROV**  **n=131 (%)** | **Co-infections** | | | | |
| --- | --- | --- | --- | --- | --- | --- | --- | --- |
|  |  |  |  | **OROV**  **n=82 (%)** | **OROV/DENV n= 36 (%)** | **OROV/ZIKV n= 9 (%)** | **OROV/CHIKV**  **n= 1 (%)** | **Others**  **N= 3 (%)** |
| February | 1 (0.2) | 0 (0.0) | 1 (0.8) | 1 (1.21) | 0 (0.0) | 0 (0.0) | 0 (0.0) | 0 (0.0) |
| March | 6 (1.2) | 5 (1.4) | 1 (0.8) | 1 (1.21) | 0 (0.0) | 0 (0.0) | 0 (0.0) | 0 (0.0) |
| April | 14 (2.8) | 9 (2.5) | 5 (3.8) | 1 (1.21) | 4 (11.1) | 0 (0.0) | 0 (0.0) | 0 (0.0) |
| May | 95 (19.2) | 61 (16.7) | 34 (26.0)* | 25(30.5)* | 5 (13.9) | 3 (33.3) | 1 (100%) | 1 (33.3) |
| June | 265 (53.4) | 199 (54.5) | 66 (50.4) | 38 (46.3) | 20 (55.5) | 5 (55.5) | 0 (0.0) | 2 (66.7) |
| July | 109 (22.0) | 86 (23.6) | 23 (17.6) | 15 (18.3) | 7 (19.4) | 1 (11.1) | 0 (0.0) | 0 (0.0) |
| August | 4 (0.8) | 4 (1.1) | 0 (0.0) | 0 (0.0) | 0 (0.0) | 0 (0.0) | 0 (0.0) | 0 (0.0) |
| September | 2 (0.4) | 1 (0.3) | 1 (0.8) | 1 (1.21) | 0 (0.0) | 0 (0.0) | 0 (0.0) | 0 (0.0) |
|  |  |  |  |  |  |  |  |  |
| *χ^2^*-Test, *p*-value | 0.616 |  | 0.045 | 0.016 | 0.861 | ND | ND | ND |
| F-test, *p*-value |  |  | 0.032 | 0.011 |  |  |  |  |

**Table S2.** Analysis of the distribution of clinical symptoms. The table is symmetric on the diagonal line. The numbers correspond to the *p*-values associated with χ^2^-Test, no significant differences (*p* >0.05). ND, not determined because there are many cells with counts less than 5. *Others correspond to infection cases by OROV+DENV+ZIKV and OROV+DENV+CHIKV.

|  |  |  |  |  | **Co-infections** | | | | |
| --- | --- | --- | --- | --- | --- | --- | --- | --- | --- |
|  |  | **Total** | **Negatives** | **OROV** | **OROV** | **OROV/DENV** | **OROV/ZIKV** | **OROV/CHIKV** | **Others*** |
|  |  | **n=496 (%)** | **n= 365 (%)** | **n=131 (%)** | **n=82 (%)** | **n= 36 (%)** | **n= 9 (%)** | **n= 1 (%)** | **n= 3 (%)** |
|  | **Total**  **n=496 (%)** |  | 0.999 | 0.881 | 0.857 | 0.987 | 0.988 | ND | ND |
|  | **Negatives**  **n= 365 (%)** | 0.999 |  | 0.630 | 0.660 | 0.967 | 0.984 | ND | ND |
|  | **OROV**  **n=131 (%)** | 0.881 | 0.630 |  | 1.000 | 0.998 | 0.996 | ND | ND |
| **Co-infections** | **OROV**  **n=82 (%)** | 0.857 | 0.660 | 1.000 |  | 0.982 | 0.985 | ND | ND |
|  | **OROV/DENV**  **n=36 (%)** | 0.987 | 0.967 | 0.998 | 0.982 |  | 0.999 | ND | ND |
|  | **OROV/ZIKV**  **n= 9 (%)** | 0.988 | 0.984 | 0.996 | 0.985 | 0.999 |  | ND | ND |
|  | **OROV/CHIKV**  **n= 1 (%)** | ND | ND | ND | ND | ND | ND |  | ND |
|  | **Others***  **N= 3 (%)** | ND | ND | ND | ND | ND | ND | ND |  |

**Table S3.** Positive cases of OROV by age and symptomatology. χ^2^-test, total positive cases vs positive cases by age.

| **CLINICAL SYMPTOMS** | **OROV**  **n=131 (%)** | **Age (years old)** | | | | | |
| --- | --- | --- | --- | --- | --- | --- | --- |
|  |  | **< 5**  **n=8 (%)** | **5 - 11**  **n=26 (%)** | **12 - 17**  **n=13 (%)** | **18 - 39**  **n=33 (%)** | **40 - 59**  **n=22 (%)** | **≥ 60**  **n=29 (%)** |
| Headache | 112 (85.5) | 6 (75.0) | 22 (84.6) | 10 (76.9) | 29 (87.9) | 20 (90.9) | 25 (86.2) |
| Myalgia | 106 (80.9) | 2 (25.0) | 20 (76.9) | 10 (76.9) | 29 (87.9) | 20 (90.9) | 25 (86.2) |
| Arthralgia | 95 (72.5) | 2 (25.0) | 16 (61.5) | 7 (53.8) | 26 (78.8) | 18 (81.8) | 26 (89.7) |
| Hyporexia | 89 (67.9) | 6 (75.0) | 15 (57.7) | 8 (61.5) | 23 (69.7) | 17 (77.3) | 20 (69.0) |
| Retroocular pain | 70 (53.4) | 2 (25.0) | 12 (46.2) | 4 (30.8) | 24 (72.7) | 14 (63.6) | 14 (48.3) |
| Low back pain | 66 (50.4) | 0 (0.0) | 4 (15.4) | 6 (46.2) | 20 (60.6) | 18 (81.8) | 18 (62.1) |
| Nausea / Vomiting | 62 (47.3) | 3 (37.5) | 9 (34.6) | 8 (61.5) | 18 (54.5) | 13 (59.1) | 11 (37.9) |
| Odynophagia | 48 (36.6) | 4 (50.0) | 14 (53.8) | 7 (53.8) | 9 (27.3) | 7 (31.8) | 7 (24.1) |
| Acne | 25 (19.1) | 1 (12.5) | 7 (26.9) | 2 (15.4) | 8 (24.2) | 5 (22.7) | 2 (6.9) |
|  |  |  |  |  |  |  |  |
| *χ^2^*-Test, *p*-value |  | > 0.10 | 0.313 | 0.908 | 0.963 | 0.968 | 0.715 |
